# Association of pre- and postoperative delirium with functional status at discharge after hip fracture: findings from the Gruppo Italiano di Ortogeriatria (GIOG 2.0) study

**DOI:** 10.1007/s41999-026-01444-8

**Published:** 2026-03-13

**Authors:** Maria Cristina Ferrara, Francesca Remelli, Caterina Trevisan, Federico Triolo, Elena Tassistro, Antonella Zambon, Chukwuma Okoye, Elena Pinardi, Alice Margherita Ornago, Alberto Finazzi, Luca Tinelli, Wenxiang Guo, Eleonora Cucini, Elena Page, Maria Grazia Valsecchi, Paolo Mazzola, Giuseppe Castoldi, Chiara Mussi, Monica Pizzonia, Paola Cena, Giuseppe Sergi, Andrea Ungar, Raffaele Antonelli Incalzi, Stefano Volpato, Giuseppe Bellelli

**Affiliations:** 1 School of Medicine and Surgery, University of Milano-Bicocca, Piazza dell’Ateneo Nuovo, 1, 20126 Milan, Italy; 2 Centro Studi Dipartimentale sulla Medicina della complessità e Cure Palliative Virgilio Floriani, University of Milano-Bicocca, Monza, Italy; 3 Department of Medical Science, University of Ferrara, Ferrara, Italy; 4 Aging Research Center - Karolinska Institutet, Stockholm, Sweden; 5 Bicocca Center of Bioinformatics, Biostatistics and Bioimaging (B4 Centre), School of Medicine and Surgery, University of Milano-Bicocca, Monza, Italy; 6 Biostatistics and Clinical Epidemiology, Fondazione IRCCS San Gerardo dei Tintori, Monza, Italy; 7 Department of Statistics and Quantitative Methods, University of Milano-Bicocca, Milan, Italy; 8 Biostatistics Unit, IRCCS Istituto Auxologico Italiano, Milan, Italy; 9 Acute Geriatric Unit – IRCCS San Gerardo dei Tintori Foundation, Monza, Italy; 10 Department of Medicine and Surgery, University of Genova, Genoa, Italy; 11 Orthopedics and Traumatology Unit - ASST della Brianza - P.O. Carate Brianza, Carate Brianza, Italy; 12 Department of Biomedical, Metabolic and Neural Sciences, University of Modena and Reggio Emilia, Modena, Italy; 13 Orthogeriatric Unit, IRCCS Ospedale Policlinico San Martino, Genoa, Italy; 14 Orthogeriatric Unit, Santa Croce e Carle Hospital, Cuneo, Italy; 15 Acute Geriatric Unit, University Hospital of Padova, Padua, Italy; 16 Department of Geriatrics, Careggi University Hospital, University of Florence, Florence, Italy; 17 Acute Geriatric Unit, Campus Bio-medico University, Rome, Italy; 18 Policlinico S.Orsola-Malpighi, Bologna, Milan, Italy; 19 Azienda USL Toscana centro, Florence, Italy; 20 Galliera Hospital, Genoa, Italy; 21 Azienda Ospedaliera SS. Annunziata, Cosenza, Italy; 22 Ospedale Ca’ Foncello AULSS 2, Treviso, Italy

**Keywords:** Hip fracture, Orthogeriatric, Delirium, Functional status, Older adults

## Abstract

**Purpose:**

Evidence on how the timing of delirium onset influences functional outcomes after hip fracture is scarce, yet is crucial for informing service planning and optimizing orthogeriatric care pathways. We therefore examined the association between delirium occurring at distinct perioperative phases and functional status at discharge in older patients undergoing hip fracture surgery.

**Methods:**

This multicenter prospective cohort study included patients aged ≥ 65 years who underwent hip fracture surgery between July 2019 and June 2024 at 12 Italian orthogeriatric centers. Delirium was assessed with the 4AT from the day before surgery through postoperative day three**.** Poor functional status at discharge was defined as a Cumulated Ambulation Score ≤ 2. Associations of preoperative, postoperative, and preoperative + postoperative delirium with poor functional status were examined using multivariable logistic regression adjusted for age, sex, comorbidity, pre-fracture activities of daily living, pre-fracture ambulation, and time-to-surgery > 48 h.

**Results:**

A total of 1492 patients were included (median age 84 years, Q1–Q3: 79–89; 76.9% female). Delirium occurred in 444 (29.8%) patients: 34 (2.3%) had preoperative delirium, 182 (12.2%) postoperative delirium, and 228 (15.3%) both. The combined presence of preoperative and postoperative delirium was significantly associated with higher odds of poor functional status at discharge (Odds Ratio: 1.57; 95% Confidence Interval: 1.13–2.20) versus no delirium.

**Conclusion:**

Delirium persisting from the preoperative to the postoperative phase was associated with poor functional status at discharge. Systematic assessment of delirium in both phases may help identify older hip fracture patients at increased risk of poor functional outcome.

**Graphical abstract:**

**Supplementary Information:**

The online version contains supplementary material available at 10.1007/s41999-026-01444-8.

## Key summary points

**Aim:**

To investigate how delirium occurring at distinct perioperative phases affects functional status at discharge in older adults undergoing hip fracture surgery.

**Findings:**

Nearly 30% of 1492 patients experienced delirium. Only persistent pre- and postoperative delirium (PRE-D + POD, 15.3%) was associated with a significantly higher risk of poor functional status at discharge.

**Message:**

Systematic assessment of delirium before and after surgery can help identify high-risk patients for poor functional outcomes.

**Supplementary Information:**

The online version contains supplementary material available at 10.1007/s41999-026-01444-8.

## Graphical abstract

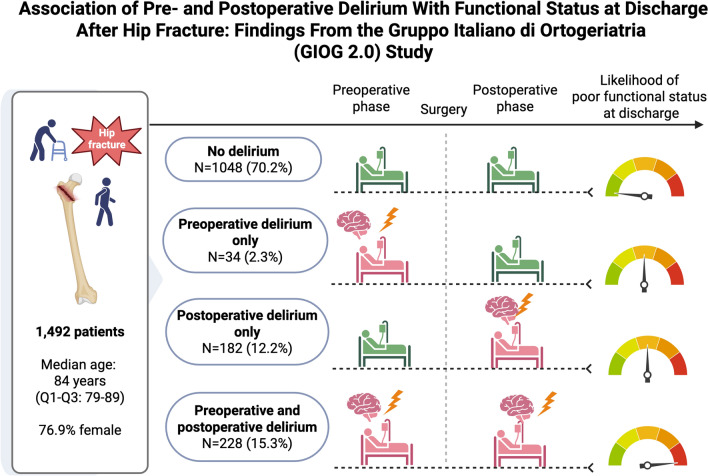

## Introduction

Hip fracture remains a major cause of residual disability in older population, despite advances in surgical techniques and perioperative care [1, 2]. Nearly half of patients are unable to walk one month after surgery, and an even higher proportion remain functionally impaired at four months [2–4]. Recovery after hip fracture is strongly influenced by key determinants, including pre-fracture functional status, cognitive function, and the early resumption of postoperative ambulation [3–7].

Delirium, an acute neuropsychiatric disorder characterized by impaired attention and awareness, fluctuating course, and global cognitive dysfunction [8], is among of the most frequent medical complications of hip fracture, affecting approximately one in four older adults [9–11]. Its duration varies considerably, ranging from a few days in most cases to several months in others [12, 13].

Delirium has been linked with several adverse outcomes, including functional decline, after a wide range of acute conditions [14, 15]. Specifically, postoperative delirium after hip fracture has been consistently associated with poorer functional status at discharge [4] and a reduced likelihood of regaining functional independence within 120 days [5]. Recent evidence in hip fracture field indicates that delirium frequently begins before surgery and may persist postoperatively [16], and that delirium at admission is associated with higher mortality and lower probability of returning home within 30 days [17]. However, it remains unclear how the timing of delirium onset affects short-term functional recovery—specifically, whether delirium occurring preoperatively only (PRE-D), postoperatively only (POD), or persisting across both phases (PRE-D + POD) differentially influences functional status at discharge in older adults with hip fracture. We hypothesized that patients with delirium persisting from the preoperative to the postoperative phase may be more vulnerable to functional decline than those with isolated PRE-D or POD.

This study therefore aimed to investigate the association between delirium at distinct perioperative phases (i.e. PRE-D, POD, and PRE-D + POD) and functional status at discharge in older patients undergoing hip fracture surgery, using data from the Gruppo Italiano di Ortogeriatria (GIOG) network.

## Methods

*Study population.* Data were prospectively collected between July 2019 and January 2024 within the GIOG 2.0 study, designed to investigate key aspects of hip fracture management among older adults in Italy [18, 19]. GIOG 2.0 is a multicenter, prospective study enrolling patients aged ≥ 65 years consecutively admitted for hip fracture to 12 orthogeriatric centers participating in the “Gruppo Italiano di OrtoGeriatria” (GIOG) network. The study protocol was approved by the Ethics Committee Brianza Institutional Review Board on April 12th, 2019. Written informed consent was obtained either from the patients themselves or their legally authorized representatives/proxies, in accordance with applicable regulations. Reporting follows the STROBE guidelines for observational studies.

*Sociodemographic and medical data.* At each center, attending physicians collected patient sociodemographic and clinical information at admission using standardized assessment forms and medical records. Baseline data included age, sex, pre-fracture living arrangement, pre-fracture ambulation level (Standardized Audit of Hip Fracture In Europe, SAHFE [20]), nutritional status (Mini Nutritional Assessment short-form [21]), comorbidity burden (Charlson Comorbidity Index [22]) pre-fracture diagnosis of dementia, pre-fracture independence in the activities of daily living (Katz index for Basic Activities of Daily Living, BADLs [23], and number of pre-fracture medications. Fracture type (intracapsular vs. extracapsular), time-to-surgery (≤ 48 vs. > 48 h [24]) were also recorded, as well as in-hospital complications (defined as the occurrence of at least one of the following events during the index admission: pneumonia, urinary tract infection, sepsis, skin/soft-tissue infection, myocardial infarction, acute heart failure, stroke, pulmonary embolism, or major bleeding/deep haematoma).

*Exposure.* Delirium was assessed using the 4A’s Test (4AT) [25], administered by the attending physicians once daily at each participating center from the day before surgery through postoperative day three. The 4AT is a brief, user-friendly delirium screening tool (sensitivity and specificity 88%) that requires no special training [26–28]. Scores range from 0 to 12 points, with a score ≥ 4 indicating possible delirium (with or without cognitive impairment). At each center, a senior geriatrician with expertise in delirium was responsible for reviewing all 4AT assessments, and confirming or excluding the final diagnosis of delirium, with full access to patients’ clinical records (medical and nursing notes, medication charts, laboratory tests, etc.), based on the Diagnostic and Statistical Manual of Mental Disorders, Fifth Edition criteria [8]. Patients with delirium occurring exclusively in the preoperative phase, exclusively in the postoperative phase, or in both phases, were classified as PRE-D, POD, and PRE-D + POD, respectively. All remaining patients were classified as not delirious (“non-Del”).

*Outcome*. The primary outcome was poor functional status at hospital discharge, defined as a Cumulated Ambulation Score [29] ≤ 2. The Cumulated Ambulation Score assesses independence in three essential activities: getting in/out of bed, rising from a chair, and walking. Each activity is scored 0–2 points: 2 if completed independently, 1 if assistance is required, and 0 if the task cannot be performed even with support. Higher scores indicate greater independence.

### Statistical analysis

Pseudo-anonymized data from all centers were entered into a centralized electronic case-report form with predefined data fields within a secure REDCap Cloud database, and all statistical analyses were performed by the coordinating center. Continuous variables are presented as medians with Quartile 1 and Quartile 3 (Q1–Q3), given their non-normal distribution, and categorical variables as counts and percentages. Descriptive group comparisons were performed using the Kruskal-Wallis test for continuous variables and the Chi-square test for categorical variables, with post-hoc pairwise comparisons adjusted by Bonferroni correction. Associations between delirium groups and poor functional outcome at discharge were estimated using multivariable logistic regression, adjusting for age, sex, comorbidity burden, pre-fracture BADLs, pre-fracture ambulation level, and time-to-surgery > 48 h. Delirium occurrence was entered as a single categorical multi-level exposure variable, with “no delirium” as the reference category. Confounders were selected based on clinical practice and current literature [30–34]. Associations were expressed as Odds Ratios (ORs) with 95% Confidence Intervals (95% CIs), and statistical significance was defined as a two-sided *p*-value < 0.05. All assumptions for logistic regression were tested and met. This primary analysis was conducted as complete-case analyses for all covariates (final analytic sample n = 1492). Patients with complete data on exposure and outcome but missing values in one or more covariates (age, sex, comorbidity burden, pre-fracture BADLs, pre-fracture ambulation level, and time-to-surgery > 48 h) were included in a sensitivity analysis using multiple imputation with a Fully Conditional Specification approach (n = 2048). Finally, another sensitivity analyses was performed including pre-fracture dementia as an additional covariate. Statistical analyses were performed using SAS, version 9.4 (SAS Institute, Inc., Cary, NC) and SPSS 26.0 (IBM Corp.).

## Results

### Study population

**Fig. 1:**
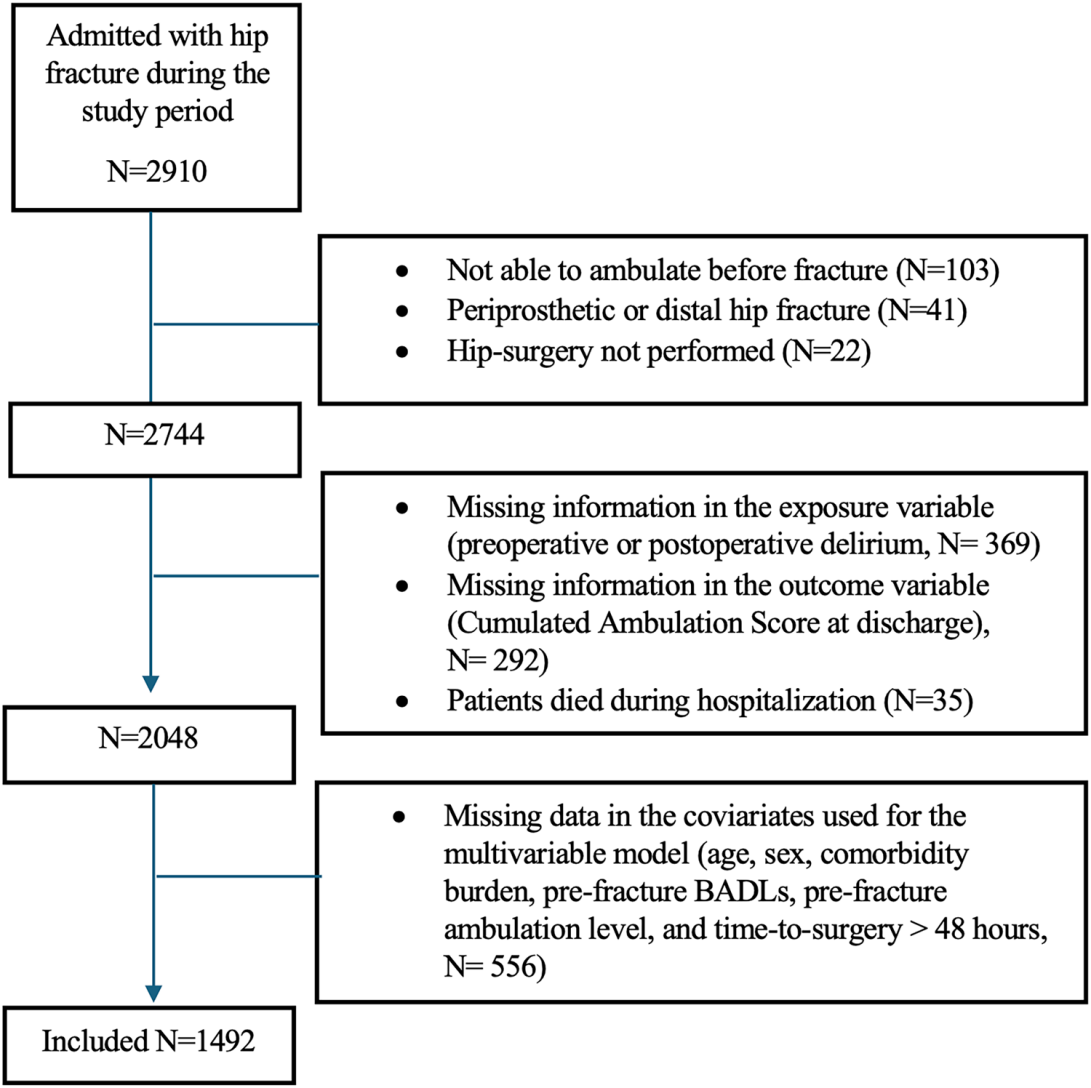
Flowchart of the study

Figure 1 shows the study flowchart. After excluding patients unable to walk before hip fracture, those with periprosthetic or distal fractures, those who did not undergo surgery, those who died during hospitalization, and those with missing data on exposure (delirium), outcome (Cumulated Ambulation Score), or covariates (age, sex, comorbidity burden, pre-fracture BADLs, pre-fracture ambulation level, and time-to-surgery > 48 h), 1,492 patients were included (median age 84 years [Q1–Q3: 79–89]; 76.9% female). Delirium was absent in 1,048 patients (“non-Del”, 70.2%), while 34 (2.3%) had PRE-D only, 182 (12.2%) had POD only, and 228 (15.3%) experienced PRE-D + POD.

### Baseline characteristics

Online Resource Table S1 summarizes the characteristics of the study population, which did not meaningfully differ from those of the entire source population [data not shown]: 281 patients (18.8%) lived at home alone and 587 (39.3%) walked independently before fracture. The median Mini Nutritional Assessment short-form score was 11 (Q1–Q3: 9–12), indicating an overall risk of malnutrition, and the median Charlson Comorbidity Index was 5 (Q1–Q3: 4–7), suggesting moderate comorbidity. Nearly one-third of patients had pre-fracture diagnosis of dementia. Extracapsular fractures occurred in 777 patients (52.1 %). Surgery ≤ 48 h was performed in 1007 patients (67.5%), and in-hospital complications occurred in 526 (35.3%). The median length of hospital stay was 10 (Q1-Q3: 8-13) days, and one-third of patients were discharged home. Overall, 35.7% of the patients had poor functional status at discharge (Cumulated Ambulation Score ≤ 2).

Baseline characteristics according to delirium groups are reported in Table 1. Patients with delirium were older, had higher comorbidity, and worse nutritional and functional status compared with the “non-Del” group. Median age increased from 83 years (Q1–Q3: 77–88) in non-Del to 85 years in PRE-D and POD, and 87 years (Q1–Q3: 84–92) in PRE-D + POD (overall *p* < 0.001). Pre-fracture BADLs declined from a median of 5 (Q1–Q3: 4–6) in non-Del to 4 (Q1–Q3: 2–5) in PRE-D, and 2 (Q1–Q3: 1–4) in PRE-D + POD (*p* < 0.001), while independent ambulation dropped from 48.2% in non-Del to around 20% in PRE-D and POD, and 13.2% in PRE-D + POD (*p* < 0.001). Therefore, the PRE-D + POD group included the oldest and most functionally dependent patients, with the highest prevalence of dementia (75.9%; *p* < 0.001), while patients in the PRE-D and POD groups exhibited intermediate characteristics between the “non-Del” and PRE-D + POD groups. Poor functional status at discharge was significantly more common in the PRE-D + POD group (56.6%) than in the “non-Del” (30.8%) and POD (36.3%) groups (*p* < 0.001).

**Table 1:** Characteristics of the study population by perioperative delirium (N = 1492)

Variables	Non-Del^a^ N = 1048 (70.2%)	PRE-D^b^ N = 34 (2.3%)	POD^c^ N = 182 (12.2%)	PRE-D + POD^d^ N = 228 (15.3%)	*p*-value
Age	83 (77–88)	85 (83–90)	85 (81–90)*^j^	87 (84–92)*^j^*^l^	< 0.001
Female sex	810 (77.3)	25 (73.5)	130 (71.4)	183 (80.3)	0.185
Living arrangement:					< 0.001
At home alone	221 (21.1)	4 (11.8)	32 (17.6)	24 (10.5)*^j^	
At home with caregiver	794 (75.8)	27 (79.4)	143 (78.6)	183 (80.3)	
Nursing home	32 (3.1)	3 (8.8)	7 (3.8)	21 (9.2)	
Pre-fracture BADLs^e^	5 (4–6)	4 (2–5)*^j^	5 (3–6)*^j^	2 (1–4)*^j^*^k^*^l^	< 0.001
Pre-fracture ambulation level (SAHFE^f^):					< 0.001
Independently	505 (48.2)	7 (20.6)*^j^	45 (24.7)^*j^	30 (13.2)^*j*l^	
One or two-aids outdoor	268 (25.6)	6 (17.6)	56 (30.8)	37 (16.2)	
Only in-door	275 (26.2)	21 (61.8)	81 (44.5)	161 (70.6)	
MNA-sf^g^	11 (10–13)	10 (9–11)*^j^	11 (9–12)*^j^	10 (8–11)*^j^*^l^	< 0.001
CCI^h^	5 (4–6)	6 (5–7)*^j^	6 (4–7)*^j^	6 (5–8)*^j^*^l^	< 0.001
Dementia	181 (17.3)	19 (55.9)*^j^	76 (41.8)*^j^	173 (75.9)*^j^*^l^	< 0.001
Number of pre-fracture medications	5 (3-6)	6 (4–8)	5 (3–6)	5 (3–6)	0.035
Extracapsular fracture	539 (51.4)	19 (55.9)	102 (56)	117 (51.3)	0.672
Time-to-surgery ≤ 48 h	698 (66.6)	23 (67.6)	127 (69.8)	159 (69.7)	0.721
In-hospital complications	332 (31.7)	13 (38.2)	87 (47.8)*^j^	94 (41.2)*^j^	< 0.001
Length of stay (days)	10 (8–12)	10 (7–12)	10 (8–13)	9 (7–13)	0.254
Discharge destination:					< 0.001
Home	359 (34.3)	8 (23.5)	54 (29.7)	73 (32.0)	
Rehabilitation setting	622 (59.4)	20 (58.9)	107 (58.8)	120 (52.6)	
Nursing home or other acute medical ward	65 (6.2)	6 (17.6)	21 (11.5)	35 (15.3)*^j^	
CAS^i^ at discharge ≤ 2	323 (30.8)	14 (41.2)	66 (36.3)	129 (56.6)*^j^*^l^	< 0.001

Data are presented as median (IQR) or n (%)

^a^Non-Del, No delirium

^b^PRE-D, Preoperative delirium only

^c^POD, Postoperative delirium only

^d^PRE-D + POD, Preoperative and postoperative delirium

^e^BADLs, Basic Activities of Daily Living

^f^SAHFE, Standardized Audit of Hip Fracture in Europe

^g^MNA-sf, Mini-Nutritional Assessment short-form

^h^CCI, Charlson Comorbidity Index

^i^CAS, Cumulated Ambulation Score

Post-hoc pairwise comparisons (significant after Bonferroni correction): *^j^ = significant difference compared to non-Del; *^k^ = significant difference compared to PRE-D; *^l^ = significant difference compared to POD

### Association between delirium and functional outcome

Table 2 shows the results of multivariable logistic regression. After adjusting for confounders, PRE-D + POD was significantly associated with poor functional status at discharge (OR = 1.57; 95% CI: 1.13–2.20). PRE-D only and POD only were not significantly associated with the outcome, but these results should be interpreted with caution given the relatively small number of patients in these groups. Limited pre-fracture ambulation was also associated with higher odds of poor functional outcome (outdoor with aids: OR = 2.22, 95% CI: 1.61–3.06; indoor only: OR = 2.38, 95% CI: 1.69–3.35). Conversely, higher pre-fracture BADLs were independently associated with lower odds of poor functional outcome (OR = 0.86; 95% CI: 0.80–0.93).

**Table 2:** Multivariable logistic regression of the association between perioperative delirium and poor functional status at discharge (N = 1492)

Variables	OR^c^ (CI 95%^d^)	*p*-value
*Delirium occurrence*		
Non-Del (no delirium)	Ref	–
PRE-D (preoperative delirium only)	1.03 (0.50–2.13)	0.928
POD (postoperative delirium only)	0.96 (0.68–1.36)	0.816
PRE-D + POD (preoperative + postoperative delirium)	1.57 (1.13–2.20)	0.008
Age (per one year increase)	1.01 (0.99–1.03)	0.302
*Sex*		
Male	Ref	–
Female	1.23 (0.93–1.62)	0.153
Charlson Comorbidity Index (per one-point increase)	1.01 (0.95–1.06)	0.834
Pre-fracture BADLs^a^ (per one-point increase)	0.86 (0.80–0.93)	< 0.001
*Pre-fracture ambulation level (SAHFE*^*b*^*)*		
Independently	Ref	–
One or two-aids outdoor	2.22 (1.61–3.06)	< 0.001
Only in-door	2.38 (1.69–3.35)	< 0.001
*Time-to-surgery*		
≤ 48 h	Ref	–
> 48 h	1.06 (0.83–1.35)	0.632

^a^BADLs, Basic Activities of Daily Living

^b^SAHFE, Standardized Audit of Hip Fracture in Europe

^c^OR, Odds Ratio

^d^CI 95%, Confidence Interval 95% (inferior limit-superior limit)

### Sensitivity analyses

As reported in the Supplementary Material (Online Resource Table S2), results from the imputed sensitivity analysis were consistent with the complete-case findings. Results were confirmed also after including pre-fracture dementia as an additional covariate (Online Resource Table S3).

## Discussion

In this large cohort study of older patients with hip fracture, persistent delirium spanning from pre- to postoperative phases (PRE-D+POD) was associated with a 57% increased risk of poor functional outcome at discharge compared to patients without delirium, while no statistically significant association was observed for patients with isolated PRE-D or POD. Pre-fracture functional status, specifically BADL performance and ambulation level, also emerged as independent determinants of functional outcome in this population.

To our knowledge, this is the first study to examine how delirium occurring at distinct perioperative phases affects functional status at discharge in hip fracture patients.

The finding that PRE-D + POD, and not isolated PRE-D or POD, is associated with poor functional outcome suggests that studies evaluating only POD may have overlooked the contribution of undetected preoperative delirium, and vice versa for studies evaluating only PRE-D. Previous research indicates that up to 20% of older patients experience delirium before surgery [17, 35], which in turn increases the risk of postoperative delirium [16, 36]. Our study’s higher prevalence of PRE-D + POD compared with isolated PRE-D or POD supports this notion. Moreover, a retrospective case-control study considering PRE-D and POD as separate phenomena found that long-term deterioration in walking ability is more frequent in patients with PRE-D than those with POD [37]. Nevertheless, we cannot completely exclude that PRE-D or POD alone may also be associated to some extent with the adverse functional outcome, given the relatively limited statistical power in these two groups. It is also conceivable that the orthogeriatric model adopted in all participating centers may have partially attenuated the short-term functional impact of isolated POD through early mobilization and rehabilitation. Therefore, these findings should be viewed as hypothesis-generating, highlighting the importance of delirium timing (onset and persistence) and the context of care, rather than contradicting current literature.

In any case, persistent perioperative delirium may identify a particularly vulnerable subgroup less able to cope with fracture- and surgery-related stress. In this perspective, delirium spanning both pre- and postoperative periods can be viewed as a clinical marker of both higher baseline vulnerability (including underlying cognitive impairment and frailty) and more severe and/or prolonged brain dysfunction across the perioperative course. Patients who are already delirious before surgery and remain delirious afterwards are likely to have limited physiological reserve and longer cumulative exposure to neuroinflammatory, metabolic, environmental and iatrogenic stressors related to fracture and surgery. A previous study supports the hypothesis by showing that individuals with both POD and frailty have poorer functional outcomes at discharge than those with isolated POD [4]. This vulnerability is compounded by the high prevalence of dementia in the PRE-D + POD group, consistent with evidence linking cognitive impairment to both prolonged delirium [13, 38], increased delirium severity [39], and poorer functional recovery. Supporting this concept, a study found that cognitive impairment, but not POD, was a determinant of ambulation recovery at discharge in patients with hip fracture [40]. However, that study did not include preoperative assessment of delirium, raising the possibility that observed pre-surgery cognitive impairment may have been influenced by undetected delirium [40]. Importantly, our sensitivity analysis demonstrated that PRE-D + POD remains an independent predictor of poor functional outcome even after adjusting for pre-fracture dementia.

The multifaceted pathophysiology of delirium, including neuroinflammation, brain vascular dysfunction, altered brain metabolism, neurotransmitter imbalance, and impaired neuronal network connectivity, may contribute to the development of acute cognitive and motor impairments by disrupting physiological processes [41–43]. These mechanisms are likely to be particularly relevant in patients with persistent delirium, in whom pathophysiological insults may accumulate or even act synergistically over time. Additionally, environmental and care-related factors, such as prolonged bed rest after hip fracture and the patient’s lack of collaboration in rehabilitation activities, may have negatively impacted functional outcomes [9, 10], with persistent delirium potentially interfering more strongly with rehabilitation and thereby triggering a self-perpetuating downward spiral of functional decline.

Taken together, these elements support the interpretation of PRE-D + POD as a marker of heightened vulnerability and prolonged brain dysfunction leading to poor functional outcome; however, this interpretation remains hypothesis-generating and warrants confirmation in future studies with more granular delirium phenotyping, standardized frailty and pre-fracture cognitive assessments, as well as incorporation of biological markers (e.g., neuroinflammation and neurodegeneration).

Consistent with prior evidence, low pre-fracture BADLs and limited ambulation—which are likely to act as pragmatic proxies of underlying vulnerability—strongly predicted poor functional outcomes, highlighting the critical role of baseline functional status in postoperative recovery [2, 5, 44, 45].

Overall, these findings underscore the importance of systematic delirium assessment both before and after surgery, ideally through daily evaluations from admission to discharge. Early recognition of preoperative delirium is crucial for assessing decision-making capacity and the validity of informed consent for surgery, providing an additional rationale for routine cognitive and delirium screening at admission and in the preoperative phase. Despite its clinical relevance, preoperative delirium assessment remains uncommon in clinical practice and hip fracture registries, whereas postoperative delirium is increasingly monitored as a performance indicator. The most recent reports from international hip fracture registries indicate that a substantial proportion of patients with hip fracture, ranging from one-third to over half, do not undergo delirium assessment on admission [46, 47]. In England, preoperative delirium screening in hip fracture patients is now formally mandated within the Best Practice Tariff, with reimbursement contingent on completion of screening, as specified in National Health Service England guidance, although the National Hip Fracture Database still identifies only postoperative delirium as a key performance indicator [48]. The Spanish Hip Fracture Registry has recently incorporated preoperative 4AT assessment among its recommended indicators, although data of its implementation are not yet available [49]. Implementing routine perioperative delirium screening could improve identification of high-risk patients and guide targeted interventions.

This study has several strengths, including a large hip fracture cohort from multiple orthogeriatric centers in Italy, longitudinal assessment of delirium using a validated tool from admission through three days postoperatively, and comprehensive clinical data collection during hospitalization. However, some limitations should be acknowledged: baseline sociodemographic and clinical data were incomplete for some participants (see Fig. 1 for details), although missingness appeared to be largely random, likely reflecting logistical issues in data collection; detailed evaluation of delirium duration, motor subtypes, and severity, was lacking, which limited our ability to explore the association between specific delirium characteristics and poor functional outcome; the relatively small number of patients in the PRE-D and POD groups may have reduced the precision of estimates due to random error, as the study was not specifically powered to detect outcome differences; therefore these estimates have to be considered primarily exploratory/hypothesis-generating and interpreted with caution; the absence of a standardized frailty and illness-severity measure may have led to residual confounding, which should be addressed in future studies; finally, the study was underpowered to clarify whether dementia acts as an effect modifier of delirium [14] at different perioperative phases, and the lack of detailed pre-admission cognitive data may have limited our ability to explore the interplay between delirium, dementia, and functional outcomes, which could be relevant for future research [39].

## Conclusions

This study highlights the importance of assessing both PRE-D and POD to identify older hip fracture patients at high risk of poor functional status at discharge. Systematic delirium evaluation is essential to guide targeted risk-reduction programs and improve functional outcomes in this population.

## Supplementary Information

Below is the link to the electronic supplementary material.

Supplementary file1 (DOCX 58 kb)

## Data Availability

Individual participant data cannot be made publicly available due to the sensitive nature of the personal health data collected and privacy and confidentiality reasons. However, under certain conditions, these data could be made accessible for statistical and scientific research. For further information, please contact the corresponding author.
