# Supplementary material for: Association of pre- and postoperative delirium with functional status at discharge after hip fracture: findings from the Gruppo Italiano di Ortogeriatria (GIOG 2.0) study
Source: Eur Geriatr Med. 2026 Mar 13;17(3):1419–28. doi: 10.1007/s41999-026-01444-8 (PMC13309458; doi:10.1007/s41999-026-01444-8)
Supplement: Supplementary file 1 — Supplementary file1 (DOCX 58 kb) [file 41999_2026_1444_MOESM1_ESM.docx]

**Association of Pre- and Postoperative Delirium With Functional Status at Discharge After Hip Fracture: Findings From the Gruppo Italiano di Ortogeriatria (GIOG 2.0) Study**

**Authors:** Maria Cristina Ferrara, MD*^a,b^; Francesca Remelli, MD*^c,d^; Federico Triolo, MD, PhD^d^; Caterina Trevisan, MD, PhD^c, d^; Elena Tassistro, MS, PhD^e,f^; Antonella Zambon, PhD^g,h^; Chukwuma Okoye, MD, PhD^a,i^; Elena Pinardi, MD^a,b,d^; Alice Margherita Ornago, MD^d^; Alberto Finazzi, MD^a,b^; Luca Tinelli, MD^a^; Wenxiang Guo, MD^a^; Eleonora Cucini, MD^a^; Elena Page, MD^j^; Maria Grazia Valsecchi, MS^e,f^; Paolo Mazzola, MD^a,i^; Giuseppe Castoldi, MD^k^; Chiara Mussi, MD^l^; Monica Pizzonia, MD^m^, Paola Cena, MD^n^, Giuseppe Sergi, MD^o^; Andrea Ungar, MD^p^; Raffaele Antonelli Incalzi, MD^q^, Stefano Volpato, MD^c^; Giuseppe Bellelli, MD^a,i^, on behalf of the GIOG Study Group.

1. School of Medicine and Surgery, University of Milano-Bicocca, Milan, Italy
2. Centro Studi Dipartimentale sulla Medicina della complessità e Cure Palliative Virgilio Floriani, University of Milano-Bicocca, Monza, Italy
3. Department of Medical Science, University of Ferrara, Ferrara, Italy
4. Aging Research Center - Karolinska Institutet, Stockholm, Sweden
5. Bicocca Center of Bioinformatics, Biostatistics and Bioimaging (B4 centre), School of Medicine and Surgery, University of Milano-Bicocca, Monza, Italy
6. Biostatistics and Clinical Epidemiology, Fondazione IRCCS San Gerardo dei Tintori, Monza, Italy
7. Department of Statistics and Quantitative Methods, University of Milano-Bicocca, Milan, Italy
8. Biostatistics Unit, IRCCS Istituto Auxologico Italiano, Milan, Italy
9. Acute Geriatric Unit – IRCCS San Gerardo dei Tintori Foundation, Monza, Italy
10. Department of Medicine and Surgery, University of Genova, Genoa, Italy
11. Orthopedics and Traumatology Unit - ASST della Brianza - P.O. Carate Brianza, Carate Brianza, Italy
12. Department of Biomedical, Metabolic and Neural Sciences, University of Modena and Reggio Emilia, Modena, Italy
13. Orthogeriatric Unit, IRCCS Ospedale Policlinico San Martino, Genoa, Italy
14. Orthogeriatric Unit, Santa Croce e Carle Hospital, Cuneo, Italy
15. Acute Geriatric Unit, University Hospital of Padova, Padua, Italy
16. Department of Geriatrics, Careggi University Hospital, University of Florence, Florence, Italy
17. Acute Geriatric Unit, Campus Bio-medico University, Rome, Italy

*These authors equally contributed to the work.

**Corresponding author:**

**Maria Cristina Ferrara, MD**

Geriatrician, PhD candidate in Public Health

Assistant Professor

School of Medicine and Surgery, University of Milano-Bicocca, Milan

Piazza dell'Ateneo Nuovo, 1 - 20126, Milan, ITALY

Tel: +39 3349406170

E-mail: mariacristina.ferrara@unimib.it; m.ferrara15@campus.unimib.it; mcris.ferrara@gmail.com

ORCID: orcid.org/0000-0002-7971-5943

# Supplementary materials

**Online Resource Table S1** Characteristics of the overall population (N=1,492)

**Online Resource Table S2** Sensitivity analysis of the association between perioperative delirium and poor functional status at discharge using multivariable logistic regression, with multiple imputation for missing covariates (N = 2,048)

**Online Resource Table S3** Sensitivity analysis of the association between perioperative delirium and poor functional status at discharge using multivariable logistic regression, including pre-fracture dementia as an additional covariate (N = 1,492)

**Online Resource Table S1** Characteristics of the overall population (N=1,492)

| **Variables** | **Total**  N=1492 |
| --- | --- |
| **Age** | 84  (79-89) |
| **Female sex** | 1148 (76.9) |
| **Living arrangement**: |  |
| At home alone | 281 (18.8) |
| At home with caregiver | 1147 (76.9) |
| Nursing home | 63 (4.2) |
| **Pre-fracture BADLs^a^** | 5 (3-6) |
| **Pre-fracture ambulation level (SAHFE^b^)**: |  |
| Independently | 587 (39.3) |
| One or two-aids outdoor | 367 (24.6) |
| Only in-door | 538 (36.1) |
| **MNA^c^** | 11 (9-12) |
| **CCI^d^** | 5 (4-7) |
| Dementia | 449 (30.1) |
| **Number of pre-fracture medications** | 5 (3-6) |
| **Extracapsular fracture** | 777 (52.1) |
| **Time-to-surgery ≤48 hours** | 1007 (67.5) |
| **In-hospital complications** | 526 (35.3) |
| **Length of stay** (days) | 10 (8-13) |
| **Discharge destination**: |  |
| Home | 494 (33.1) |
| Rehabilitation setting | 869 (58.2) |
| Nursing home or other acute medical ward | 127 (8.5) |
| **CAS^e^ at discharge ≤2** | 532 (35.7) |

Data are presented as median (IQR) or n (%).^a^ BADLs, Basic Activities of Daily Living; ^b^ SAHFE, Standardized Audit of Hip Fracture In Europe; ^c^ MNA-sf, Mini-Nutritional Assessment short-form; ^d^ CCI, Charlson Comorbidity Index; ^e^ CAS, Cumulated Ambulation Score.

**Online Resource Table S2** Sensitivity analysis of the association between perioperative delirium and poor functional status at discharge using multivariable logistic regression, with multiple imputation for missing covariates (N = 2,048)

| **Variables** | **OR^c^** **(CI 95%^d^)** | **p-value** |
| --- | --- | --- |
| *Delirium occurrence*  non-Del (no delirium)  PRE-D (preoperative delirium only) | ref  0.86 (0.53 – 1.40) | -  0.556 |
| POD (postoperative delirium only) | 1.00 (0.77 – 1.30) | 0.995 |
| PRE-D + POD (preoperative + postoperative delirium) | 1.34 (1.03 – 1.72) | 0.026 |
| Age (per one year increase) | 1.01 (0.99 – 1.03) | 0.038 |
| *Sex* |  |  |
| Male | ref | - |
| Female | 1.03 (0.91 – 1.16) | 0.631 |
| Charlson Comorbidity Index (per one-point increase) | 1.01 (0.95 – 1.06) | 0.834 |
| Pre-fracture BADLs ^a^ (per one-point increase) | 0.86 (0.80 – 0.93) | < .001 |
| *Pre-fracture ambulation level (SAHFE ^b^)*  Independently  One or two-aids outdoor | ref  2.22 (1.61 – 3.06) | -  < .001 |
| Only in-door | 2.38 (1.69 – 3.35) | < .001 |
| *Time-to-surgery* |  |  |
| ≤ 48 hours | ref | - |
| > 48 hours | 1.06 (0.83 – 1.35) | 0.632 |

^a^ BADLs, Basic Activities of Daily Living; ^b^ SAHFE, Standardized Audit of Hip Fracture In Europe; ^c^ OR,Odds Ratio; ^d^ CI 95%, Confidence Interval 95% (inferior limit - superior limit).

**Online Resource Table S3** Sensitivity analysis of the association between perioperative delirium and poor functional status at discharge using multivariable logistic regression, including pre-fracture dementia as an additional covariate (N = 1,492)

| **Variables** | **OR^d^** **(CI 95%^e^)** | **p-value** |
| --- | --- | --- |
| *Delirium occurrence*  non-Del (no delirium)  PRE-D (preoperative delirium only) | ref  0.98 (0.48 – 2.04) | -  0.968 |
| POD (postoperative delirium only) | 0.93 (0.65 – 1.32) | 0.682 |
| PRE-D + POD (preoperative + postoperative delirium) | 1.46 (1.03 – 2.08) | 0.034 |
| Age (per one year increase) | 1.01 (0.99 – 1.03) | 0.332 |
| *Sex* |  |  |
| Male | ref | - |
| Female | 1.22 (0.92 – 1.61) | 0.170 |
| Charlson Comorbidity Index (per one-point increase) | 1.00 (0.94 – 1.06) | 0.989 |
| Pre-fracture BADLs ^a^ (per one-point increase) | 0.87 (0.81 – 0.94) | < .001 |
| *Pre-fracture ambulation level (SAHFE ^b^)*  Independently  One or two-aids outdoor | ref  2.20 (1.59 – 3.03) | -  < .001 |
| Only in-door | 2.34 (1.66 – 3.30) | < .001 |
| *Time-to-surgery* |  |  |
| ≤ 48 hours  > 48 hours  Dementia ^c^ | ref  1.06 (0.84 – 1.35)  1.20 (0.91 – 1.59) | -  0.606  0.192 |

^a^ BADLs, Basic Activities of Daily Living; ^b^ SAHFE, Standardized Audit of Hip Fracture In Europe; ^c^ Diagnosis of dementia extrapolated by the Charlson Comorbidity Index; ^d^ OR,Odds Ratio; ^e^ CI 95%, Confidence Interval 95% (inferior limit - superior limit).
